# Selection of Novel Peptides Homing the 4T1 CELL Line: Exploring Alternative Targets for Triple Negative Breast Cancer

**DOI:** 10.1371/journal.pone.0161290

**Published:** 2016-08-22

**Authors:** Vera L. Silva, Debora Ferreira, Franklin L. Nobrega, Ivone M. Martins, Leon D. Kluskens, Ligia R. Rodrigues

**Affiliations:** CEB—Centre of Biological Engineering, Universidade do Minho, Campus de Gualtar, Braga, Portugal; University of Alabama at Birmingham, UNITED STATES

## Abstract

The use of bacteriophages to select novel ligands has been widely explored for cancer therapy. Their application is most warranted in cancer subtypes lacking knowledge on how to target the cancer cells in question, such as the triple negative breast cancer, eventually leading to the development of alternative nanomedicines for cancer therapeutics. Therefore, the following study aimed to select and characterize novel peptides for a triple negative breast cancer murine mammary carcinoma cell line– 4T1. Using phage display, 7 and 12 amino acid random peptide libraries were screened against the 4T1 cell line. A total of four rounds, plus a counter-selection round using the 3T3 murine fibroblast cell line, was performed. The enriched selective peptides were characterized and their binding capacity towards 4T1 tissue samples was confirmed by immunofluorescence and flow cytometry analysis. The selected peptides (4T1pep1 –CPTASNTSC and 4T1pep2—EVQSSKFPAHVS) were enriched over few rounds of selection and exhibited specific binding to the 4T1 cell line. Interestingly, affinity to the human MDA-MB-231 cell line was also observed for both peptides, promoting the translational application of these novel ligands between species. Additionally, bioinformatics analysis suggested that both peptides target human Mucin-16. This protein has been implicated in different types of cancer, as it is involved in many important cellular functions. This study strongly supports the need of finding alternative targeting systems for TNBC and the peptides herein selected exhibit promising future application as novel homing peptides for breast cancer therapy.

## Introduction

Breast cancer is the most frequent cancer amongst women, with an estimated 1.67 million new cases in 2012 [1]. The triple negative breast cancer (TNBC) subtype is responsible for 10 to 20% of all diagnosed breast cancers, as no specific targeted therapy exists [2, 3]. TNBC is characterized by larger tumors and higher grades, with a high metastasis rate and shorter recurrence period [4, 5]. Due to the lack of known receptors and the fact that current *in vivo* models for this type of tumor may not correctly mimic human breast cancer, the search for alternatives has intensified. The 4T1 mammary adenocarcinoma murine cell line has presented itself as a good model system as it closely resembles human TNBC. 4T1 also has the advantage of being able to be translated in orthotropic positions and in immune competent recipients for a more reliable biological system that can correctly mimic the response to targeting and therapeutics [6, 7].

The current treatment options for TNBC include surgery, radiotherapy and classical chemotherapy, but further potential therapeutics for TNBC have been reported and are reviewed elsewhere [8, 9]. Regarding targeted drug delivery for breast cancer, studies have largely centered on pre-clinical studies using anti-HER2 antibody-linked liposomal drugs. However, few reports have studied the use of peptides for targeted drug delivery in breast cancer treatment, especially in TNBC [10]. Previous studies were able to successfully identify a peptide that specifically binds, both *in vitro* and *in vivo*, brain metastasis derived from the TNBC MDA-MB-231 cell line [11]. Additionally, the peptide AP8, identified using a Ph.D.-7™ Phage Display Peptide Library, showed high homology and affinity to the αFGF receptor FGFR1, with subsequent biological activity in both breast cancer cells and vascular endothelial cells [12]. To date, only two successful studies showed the use of pH sensitive low insertion peptides (pHLIPs) and a 12-mer secreted clusterin (sCLU) binding peptide for the 4T1 cell line [13, 14], thus confirming the beneficial use of peptides for the delivery of therapeutic cargoes to advanced staged triple negative breast tumors. Given this, there is an unmet need to develop novel approaches to specifically target these cells.

The search for novel anticancer therapies has emerged and the use of peptides that target cancer cells has been a widely studied strategy to improve classical chemotherapy and enhance drug delivery systems. Peptides have become an attractive alternative, as they are easily synthesized and modified to improve stability, solubility and tissue penetration [15, 16].

Phage display has been highly successful in generating peptide ligands against a large variety of novel targets concerning solid tumors and the tumor niche, both *in vitro* and *in vivo*, unraveling the overall strength of this methodology to personalize targeted cancer treatment [17–20].

In this study, we describe the identification of two novel peptide ligands homing the 4T1 cell line. The identified peptides were characterized and their binding specificity towards the 4T1 cell line was confirmed by immunofluorescence and flow cytometry. Screening and analysis of possible targets is discussed, taking into account their future use as a new targeting system for TNBC treatment.

## Materials & Methods

### Cell culture, media and buffers

Mammary adenocarcinoma murine 4T1 cell line (kindly provided by Dr. João Nuno Moreira, from Centre for Neuroscience and Cell Biology (CNC) (University of Coimbra, Portugal)) were grown in RPMI 1640, while murine fibroblast 3T3 (kindly provided by Dr. Miguel Gama from Centre of Biological Engineering (CEB) (University of Minho, Portugal)) and MDA-MB-231 (ATCC^®^ HTB-26^™^) cells were grown in DMEM (Merck Millipore). Both media were supplemented with 1% penicillin-streptomycin (Merck Millipore) and 10% FBS (Merck Millipore). All cell lines were cultured at 37°C and 5% CO_2_. Sub-culturing was performed at a cell confluence of approximately 80%. Adherent cells were washed with PBS 1X pH 7.4 [137 mM NaCl, 2.7 mM KCl, 10 mM Na_2_HPO_4_ and 2 mM KH_2_PO_4_] solution and detached with trypsin-EDTA (Merck Millipore) before sub-culturing. All chemical reagents used were of analytical grade.

### *In vitro* phage biopanning

Peptides were selected from two commercially available random amino acid libraries from New England Biolabs (NEB), displayed on the minor coat protein III (pIII) of the M13 phage via N-terminal fusion, namely the 7-mer flanked by a pair of cysteine residues (The Ph.D.™-C7C Phage Display Peptide Library) and the 12-mer (The Ph.D.™-12 Phage Display Peptide Library), using a modified biopanning protocol, the Biopanning and Rapid Analysis of Selective Interactive Ligands (BRASIL) method [21]. Briefly, approximately 1×10^6^ cells/mL were collected, centrifuged at 250 x *g* for 10 min and the pellet suspended in 1 mL of complete DMEM medium containing 1% (w/v) bovine serum albumin (BSA, Sigma). The cell suspension was centrifuged with the same conditions and the blocking procedure was repeated 3 times. Afterwards, cells were suspended in complete DMEM medium containing 3% of BSA and kept on ice. Ten μL of the original library [C7C: 1×10^13^ plaque-forming units (PFUs) or 12-mer: 2×10^13^ PFUs] was added to the previous cell suspension and incubated on ice for 4 h.

An organic phase solution was prepared, consisting of cyclohexane:dibutyl phthalate (1:9 (v/v), FisherScientific) and about 7 mL of this solution was added to previously prepared BRASIL tubes. A 300 μL PBS droplet was formed after adding this buffer to the lower non-miscible organic phase. About 200 μL of cell suspension containing the binding library was gently inserted in the aqueous PBS droplet and the solution was centrifuged at 10,000 x *g* for 10 min. After centrifuging, the pellet containing the eluted phages was recovered and resuspended in 50 μL of Tris-HCl (10 mM pH 9.5, Sigma). In the case of eluted phages amplified between rounds with the 12-mer library, the amplification was performed by infecting an improved *E*. *coli*^+^ strain JM109. This strain contains a plasmid that compensates for low abundance of certain tRNAs, reducing amplification bias and enriching in binding sequences [22]. After amplification, the eluted phages are purified and concentrated with 20% (w/w) polyethylene glycol (PEG, Applichem) 8000/2.5 M NaCl solution. Phage titer was determined was determined using the double layer agar technique in LB agar plates [25 g/L Luria Bertani broth (Nzytech) with 20 g/L agar (Frilabo)], containing 0.25 mM IPTG (Nzytech) and 0.05 g/L X-gal (Nzytech). The number of transducing units was calculated by counting the blue plaques on the plates after 12 h at 37°C.

Both the eluted phages not amplified (from the C7C library) and those amplified between rounds (from the 12-mer library) were used for additional rounds of biopanning. A total of four selection rounds, plus a counter-selection round (CSR) with 3T3 cells, were performed. The CSR differs from the selection rounds, as the collected fraction is the aqueous phase containing the non-binding phages and not the pellet containing the eluted phages.

### Preparation of individual clones for peptide analysis

ssDNA was prepared according to a standard protocol [23], using iodide buffer [10 mM Tris–HCl, 1 mM EDTA and 4 M NaI pH 8.0 (Riedel-de Haen)], followed by ethanol precipitation. The pellet was resuspended in 30 μL of Tris-EDTA buffer [TE buffer 1X: 0.1 M Tris-base pH 8.0 and 10 mM EDTA] and the product was quantified using Nanodrop 1000 (ThermoScientific).

### PCR, DNA sequencing and insert analysis

Individual phages were isolated and their DNA was extracted and prepared for sequencing, to ascertain the displayed foreign peptide of each phage. The insert size of each clone was analyzed by PCR using the forward primer 5’- TTAACTCCCTGCAAGCCTCA-3’ and the reverse primer 5- CCCTCATAGTTAGCGTAACG -3. PCR reactions were carried out in a 20 μL reaction volume containing 2 μL of phage DNA, 10 mM KAPA dNTP mix (KapaBiosystems), 1U/μL Kapa *Taq* polymerase (KapaBiosystems) and 10 μM of each primer. The PCR reaction was performed in a MyCycler Thermal Cycler (Bio-Rad Laboratories) using the following conditions: 25 cycles of denaturation at 95°C for 30 s; annealing in the temperatures range from 45 to 70°C, for 30 s; and extension at 72°C for 30 s. The product size was assessed by 1% agarose gel electrophoresis in TAE 1X buffer (Tris-acetate EDTA Buffer) at 70 volts for 50 min. A NucleoSpin® Gel and PCR Clean-up kit (Macherey-Nagel) was used to remove residual PCR components from the above DNA products for sequencing analysis. DNA sequencing was carried out by Macrogen Inc. service, using the M13-pIII sequencing primer 5’- TTAACTCCCTGCAAGCCTCA-3’, provided with the library kits. Vector NTI software (Vector NTI Advance, version 11.5, Invitrogen) was used for the analysis of correct insertions of the selected peptides taking into account that the displayed peptides are expressed at the N-terminus of pIII, followed by a short spacer (G-G-G-S) and the wild-type pIII sequence.

### Bacteriophage labelling

Around 1×10^10^ PFUs/mL of phage:peptide 4T1pep1 and 4T1pep2 were precipitated with PEG/NaCl solution and resuspended in 0.1 M NaHCO_3_ pH 8.5 (Sigma). About 1 mg of Alexa Fluor 488 tetrafluorophenyl ester (Life Technologies) was dissolved in 0.1 mL of anhydrous DMSO (Sigma) and stored in 10 μL aliquots. One aliquot was added to each of the phage preparations and the mixtures were incubated for 1 h at room temperature with agitation. Following the reaction, bacteriophage particles were added to a sanitized Microsep Advance Centrifuge Device 10 K MWCO (Pall Corporation) and were centrifuged at 7,500 x *g* for 5 min at 4°C. This procedure allowed the purification of labeled conjugated and non-conjugated phages, as free Alexa 488 will pass through the membrane to the filtrate receiver. The upper solution was resuspended in 1 mL of PBS.

### Flow cytometry analysis

To characterize the selected peptides specificity and selectivity, phage:peptide 4T1pep1 and 4T1pep2 previously conjugated with Alexa 488 were analyzed using flow cytometry to evaluate their binding towards 4T1, 3T3 and MDA-MB-231 cells. Briefly, 1×10^5^ cells were harvested, washed in PBS and blocked using PBS solution supplemented with 3% of BSA at 4°C for 1 h. Cells were then washed with PBS with Tween 20 1X [PBST 1X: PBS and 0.1% (v/v) Tween 20 (Applichem)] and incubated with 100 μL of fluorescent phage particles. The cells were rinsed again with PBST and finally resuspended in 200 μL of PBS for flow cytometry analysis using an EC800™ flow cytometer analyzer (Sony Biotechnology). A total of 20000 events were accounted.

### Immunofluorescence microscopy

4T1 and 231 mammary cancer tissue sections embedded in paraffin were deparaffinized and rehydrated as described in [24]. To maximize antibody binding, antigen retrieval was performed by heating slides in 10 mM sodium citrate buffer pH 6.0 (Sigma) at 95°C for 20 min and slow cooling at room temperature for about 20 min.

Tissue samples were blocked using a 5% BSA solution added directly onto the specimen and incubated at room temperature for 30 min. Immunostaining was performed as previously described [25, 26] by adding 100 μL of 4T1pep1 and 4T1pep2 (1x10^10^ PFUs/mL) to the tissue for subsequent incubation overnight at 4°C. As a control, a slide with phage wild-type particles was used. Next, slides were washed 4X with Tris Buffered Saline with Tween 20 1X [TBST 1X: TBS and 0.1% (v/v) Tween 20] for 5 min and 100 μL of primary rabbit anti-fd bacteriophage antibody (Sigma) (working dilution of 1:5000 in BSA 1%) was added to all slides and incubated at 4°C overnight. The coverslips were rinsed again 4X with TBST 1X and incubated with the FITC-labeled goat anti-rabbit IgG secondary antibody (Sigma) (working dilution of 1:40 in BSA 1%) at room temperature for 2 h. After the additional washing steps with TBST 1X, the slides were stained with Vectashield® mounting media with DAPI (Vector Laboratories). The tissue slides were allowed to dry for 1 h at room temperature in the dark. Images were captured using an Olympus BX51 microscope incorporated with a high-sensitivity camera Olympus DP71 with 40X and 60X magnifications.

### Peptide analysis and homology

In order to accurately eliminate the existence of target unrelated peptides, false positives and already existing mimotopes, the identified sequences were scanned using the SAROTUP webserver software [27] and they were screened using the MimoDB database [28]. Since the currently existing sequence alignment tools are developed for long protein sequences, therefore not suitable when analyzing short peptides, the sequences were further analyzed by the BLAST algorithm for homology to proteins with known or putative cancer correlations. The query was performed using the BLAST search program (BLASTP 2.2.31+) against the *Homo sapiens* and *Mus musculus* non-redundant protein database using Blastp (BLASTp with word size of 3 and Blosum62 matrix, http://blast.ncbi.nlm.nih.gov/) [29].

### Statistical analysis

Data was expressed as the mean ± standard deviation (SD) of three independent experiments. Two-way ANOVA with Bonferroni post-test was performed using GraphPad Prism (GraphPad Prism version 6.00 for Windows) to identify differences among multiple groups, considering a significance level of 95%.

## Results

### *In vitro* biopanning and selection of novel peptides for 4T1 cells

**Fig 1:**
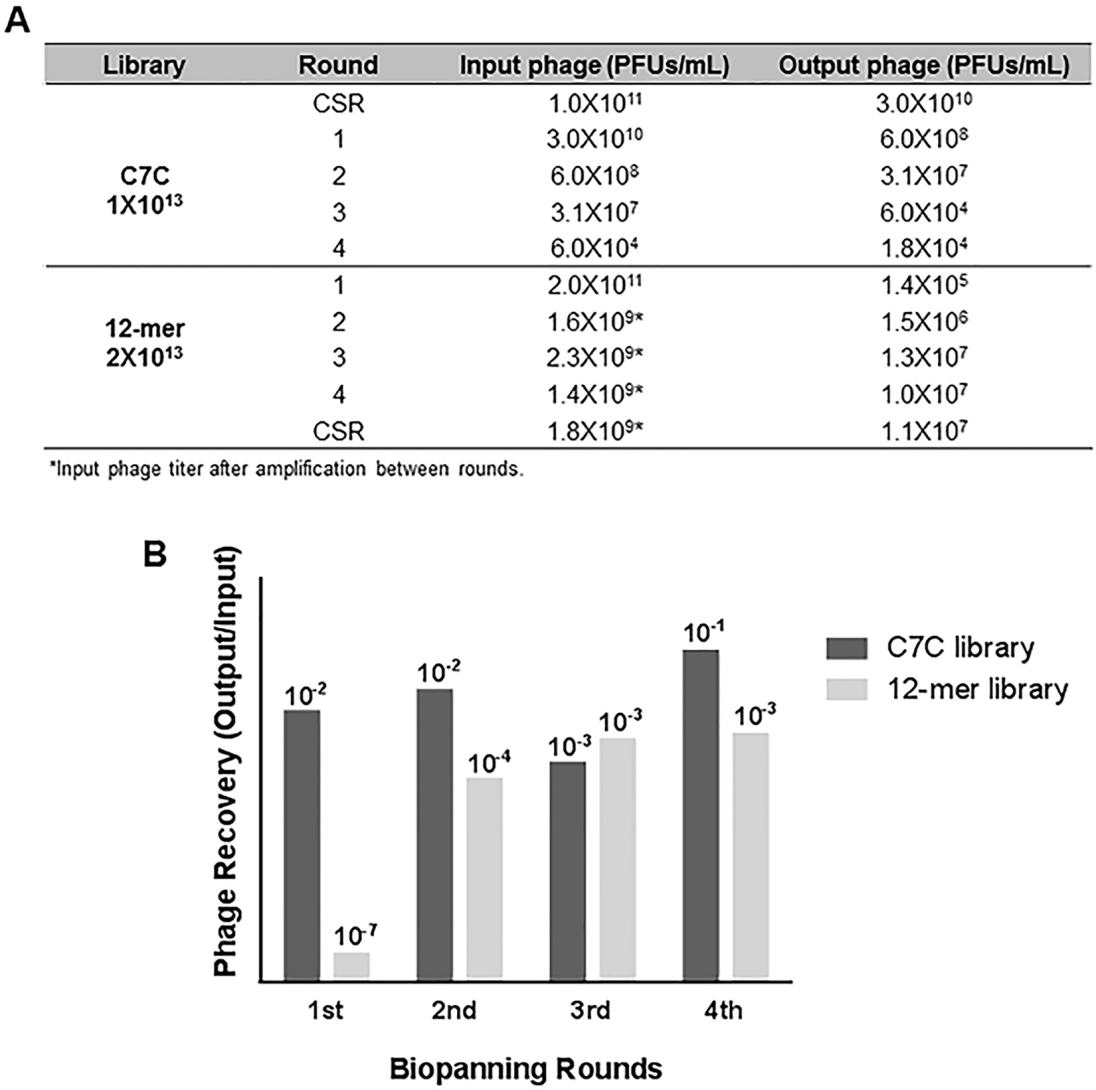
Enrichment of binding phages and phage recovery after i*n vitro* biopanning rounds against the 4T1 cell line using C7C and 12-mer libraries. (A) Enrichment of binding phages for each biopanning round. The phage titer was assessed between rounds and compared between the two modified panning procedures. (B) Phage recovery yield of internalized phages after consecutive selection step. The phage recovery of both libraries was calculated by dividing the number of recovered plaque-forming phages (output) by the input number of plaque-forming phages.

Two independent phage display experiments were performed using the BRASIL methodology. The first experiment was conducted using the C7C library starting with a CSR against the negative control 3T3 cell line, to eliminate non-specific peptides and maximize binding efficiency, followed by a total of four selection rounds against 4T1 cells. We choose not to amplify the phage particles obtained in each round because this amplification has been shown to decrease the libraries diversity [30] by enriching the pool with clones that have a growth advantage [31]. Since the phage pools between rounds were not amplified, the output of one round was the input of the next round and we could observe a phage concentration decrease from round 1 to round 4 (**Fig 1A**).

The second experiment was performed using the 12-mer library, also for a total of four selection rounds using the 4T1 cell line but in this case, the CSR with the 3T3 cells was performed after the selection rounds. Given the loss of phage populations observed during the successive biopanning rounds with the C7C library (**Fig 1A**), in these experiments with the 12-mer library, amplification between each round was conducted. The number of recovered phages after amplification is higher, leading to a high quantity of phages added to each biopanning round, hence to a higher input titer (**Fig 1A**). After titering, the phage binding efficiency of both libraries was calculated by dividing the number of recovered plaque-forming phages (output) by the input number of plaque-forming phages (phage recovering). Due to the absence of phage pool amplification between rounds in the experiments conducted with the C7C library, we can observe a very low phage recovery rate and even a decrease from round 2 to round 3 (**Fig 1B**). Nevertheless, for the 12-mer library, a 10-fold higher enrichment was observed from round 1 to round 2, followed by a subsequent plateau phase after round 3.

These results clearly show an enhanced enrichment of cell-binding phages (increase in binding efficiency) for the 4T1 cells. The enrichment varied between the libraries and a strong enrichment did not necessarily correlate with the selection of a single cell-specific phage.

### PCR amplification and DNA sequencing analysis

**Fig 2:**
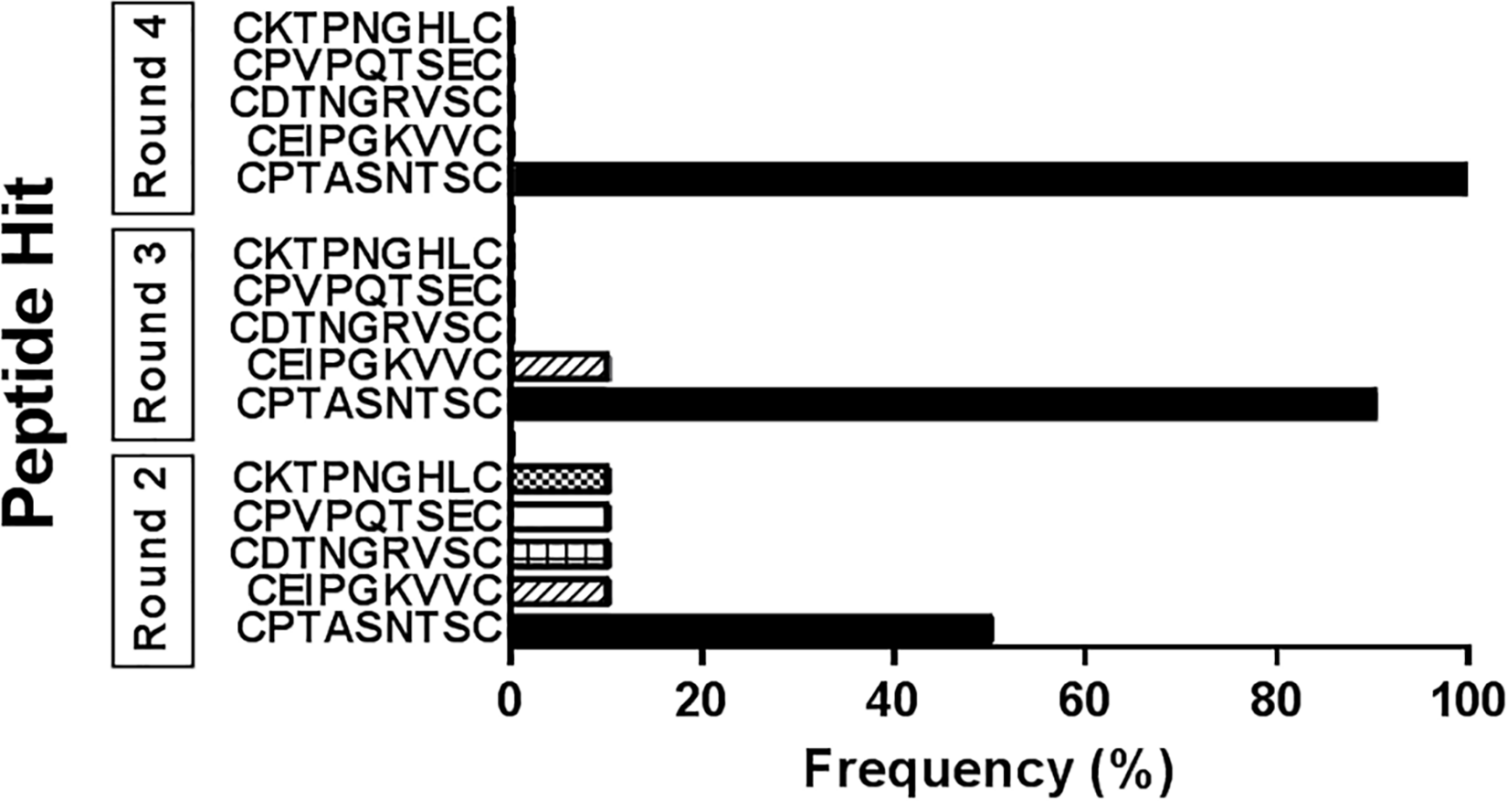
List of the most enriched phage peptides for the C7C library ranked based on their relative proportion to all PFUs collected phages after the 2^nd^, 3^rd^ and 4^th^ rounds.

After four selection rounds, individual clones were characterized by DNA sequencing. For the C7C library, a total of 22 random clones from rounds 2, 3 and 4 were individually amplified in *E*. *coli*. The DNA of each clone was isolated according to the manufacturer’s instructions [23] and the fragment containing the peptide insert was amplified by PCR. The products were analyzed by DNA agarose electrophoresis and all the clones showed a positive 329 bp-sized band (*data not shown*). Sequencing was performed for rounds 2, 3, and 4. The results showed five different peptide hits (**Fig 2**), with enrichment of the peptide CPTASNTSC over subsequent rounds. This peptide showed 50% frequency at round 2 with a 1.8 fold-enrichment at round 3. This continuous increasing proportion over other peptide hits was maintained, until 100% frequency was achieved in round 4. This data indicates that a novel peptide (4T1pep1 –CPTASNTSC) was selectively identified for 4T1 cells.

In the experiments conducted with the 12-mer library, sequencing was only performed for the last round (CSR) due to cost and time related factors, masking the analysis of different peptide hits and subsequent enrichment over rounds, as previously shown. Nonetheless, further studies were carried on, which validated the peptide selected from this library regarding its capacity to specifically bind the 4T1 cell line. In this case, a total of 12 random clones from the CSR were individually amplified in *E*. *coli*. Again, the DNA of each clone was isolated and the fragment containing the peptide insert selected from previous biopanning round was amplified by PCR. The products were analyzed by DNA agarose electrophoresis showing a positive 335 bp-sized band (*data not shown*). The sequencing results revealed the presence of a new 12 amino acid sequence peptide (4T1pep2—EVQSSKFPAHVS), which resulted from 100% homology between the clones obtained from the CSR round.

### Validation of peptide binding

**Fig 3:**
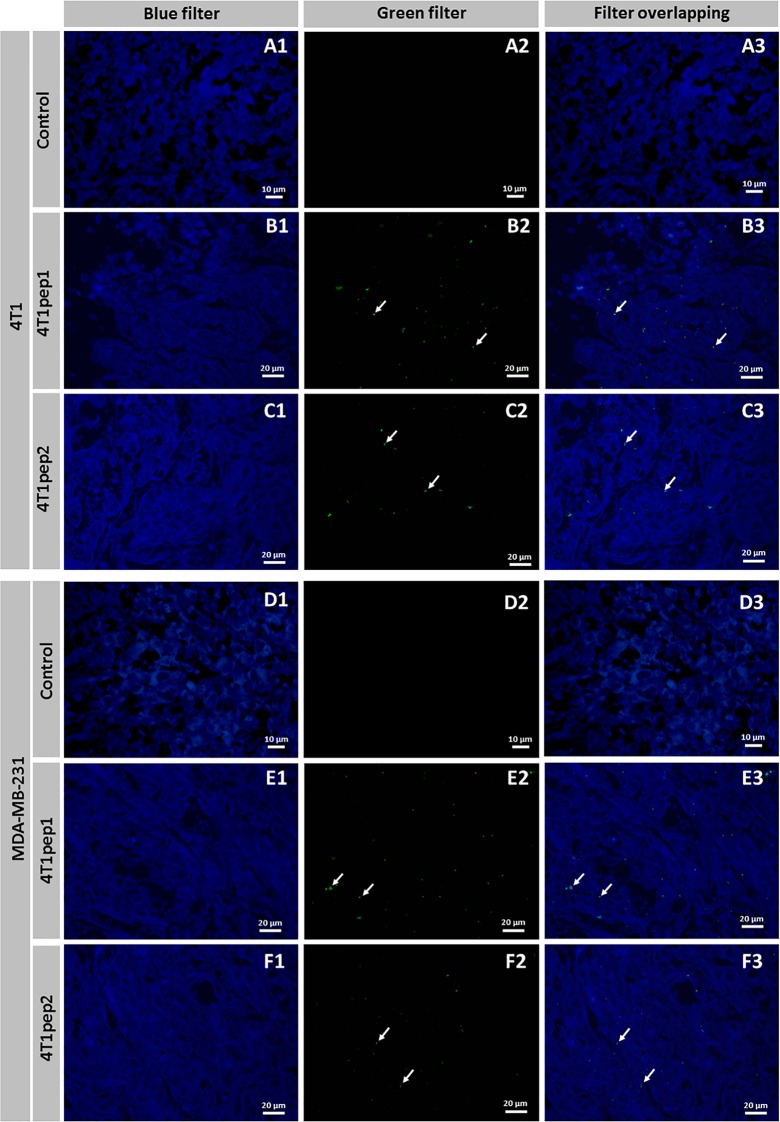
Immunofluorescence staining of 4T1 (A-C) and MDA-MB-231 (D-E) tissues with 4T1pep1 and 4T1pep2. (A1-A3) 4T1 tissue section incubated with wild-type M13 phage (negative control). (B1-B3) 4T1 tissue section incubated with M13 phage displaying 4T1pep1, stained with DAPI (B1) and FITC (B2). (C1-C3) 4T1 tissue section incubated with M13 phage displaying 4T1pep2, stained with DAPI (C1) and FITC (C2). (D1-D3) MDA-MB-231 tissue section incubated with wild-type M13 phage (negative control). (E1-E3) MDA-MB-231 tissue section incubated with M13 phage displaying 4T1pep1, stained with DAPI (E1) and FITC (E2). (F1-F3) MDA-MB-231 tissue section incubated with M13 phage displaying 4T1pep2, stained with DAPI (F1) and FITC (F2).

To confirm the binding ability of the selected peptides to 4T1 receptors, an immunofluorescence assay was performed, using a specific antibody against M13 filamentous phage. Phage particles displaying the selected peptides 4T1pep1 and 4T1pep2 were applied to 4T1 tissue sections and wild-type M13 phage was used as negative control. Additionally, 231 tissue sections were used as a human TNBC model to evaluate the possible existence of peptide species cross-reactivity and to support the translational purposes of this study (**Fig 3**).

The results showed no significant fluorescence for the negative control with both tissue sections and a bright green signal in the tissues incubated with the peptides, thus confirming that only phages displaying the selected peptides bound specifically to the tissue. Both peptides identified by phage display using different libraries were able to bind efficiently to 4T1 and MDA-MB-231 tissue sections (n = 2). The presence of fluorescent phage particles on the sample, after several washing steps, validates the specificity and binding capacity of the peptides selected by the BRASIL method.

**Fig 4:**
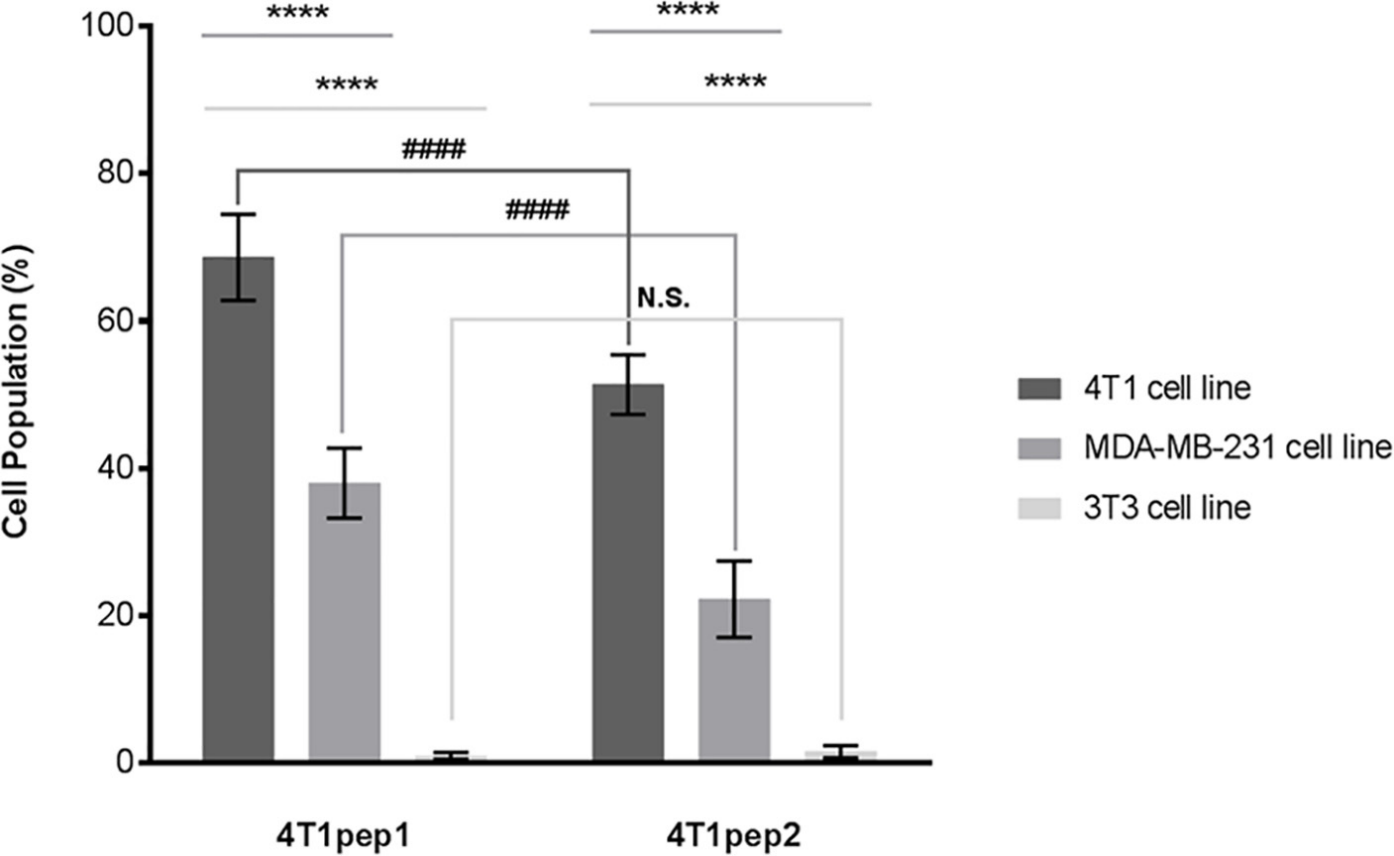
Flow cytometry results expressing the percentage of binding of the peptides 4T1pep1 and 4T1pep2 to 4T1, 3T3 and MDA-MB-231 cell lines. All data are expressed as the mean ± SD of three independent experiments. Two-way ANOVA indicates statistically significant differences within the group assessed by Bonferroni post-test and denoted as follows: ****p < 0.0001 indicates comparison between cell lines for the same peptide and ^####^*p* < 0.0001 comparison of the two peptides for the same cell line. N.S. indicates no significant difference (*p* > 0.05).

Moreover, the high selectivity of 4T1 cell line targeting peptides was confirmed by flow cytometry. About 69% of the overall cell population was bound to the 4T1pep1 (*p* < 0.0001) and around 51% to the 4T1pep2, representing 18% decreased affinity for the 4T1pep2 as compared to the 4T1pep1 **(Fig 4**). This may be due to the additional amplification step performed for the second biopanning experiment. Furthermore, no positive binding was found when the peptides were incubated with the control 3T3 cells, confirming their great specificity. The 4T1pep1 exhibited an extremely higher binding capacity (*p* < 0.0001) for the 4T1 cells as compared to the control (3T3 cells). Interestingly, a high affinity was also found for the human cell line MDA-MB-231 (*p* < 0.0001) and similar trends in the binding capacity were observed for both peptides. In summary, both peptides showed successful binding to both TNBC cell lines, therefore confirming not only their selectivity but also possible cross-reactivity amongst species.

### Bioinformatics analysis: search for candidate targets

To determine possible existence of off-target sequences, due to non-specific binding, we analyzed our sequences using SAROTUP [27]. No previous hit sequences similar to the peptides identified in this work were achieved, highlighting the novelty of the identified sequences (**Table 1**).

**Table 1:** Validation of the selected peptides through the analysis of existing target-unrelated peptides, false positives and/or mimotopes. SAROTUP freely available at http://immunet.cn/sarotup/index.html was the software used.

Analysis/Purpose	Results
TUPscan to screen for target-unrelated peptides	No motif encountered
Mimodb to screen for published mimotopes of other groups with various target peptide	No hits found, indicating that the peptide is target specific
Mimoblast to check mimotope database for peptide identity	*E*-value* too high, indicating non-reliable similarity to existing peptides

* Lowest-energy weighed score.

After four rounds of *in vitro* phage display selection, the peptide sub-population shows a collection of murine breast tumor-avid phage clones. Translated DNA sequences were analyzed by the BLAST algorithm and the identification of possible cancer-related targets by ligand homology was queried [32]. Homology of selected peptides to known proteins can sometimes be informative in identifying either ligands or possible candidates for the native cell binding ligand. Short peptide homologies to intracellular, membrane and extracellular proteins can be found, however relevancy in their cellular location and biological activity must be taken into account [33]. For each peptide sequence, top matching proteins were evaluated for percent homology, *E*-value presented and for their possible existence as membrane-bound proteins. Two interesting targets related to putative cancer proteins were obtained and are shown in **Table 2**. All proteins showed an overall identity above 60% and maximum scores superior to 15 bits with 0 gaps. The match returned variable *E*-values, but only those inferior to 10 were considered, in order to minimize the random matches in the entire protein database. Therefore, it is common that the identified peptide has little sequence similarity with its template, though they do have similar physicochemical properties and spatial organization locally.

**Table 2:** Genbank search results using NCBI protein–protein blast BLASTP 2.1.3 for the selected peptide sequences. Peptides were analyzed against *Homo sapiens* and *Mus musculus* non-redundant protein database using BLASTP for cancer related proteins (PSI-BLAST, word size of 3, Blosum62 matrix and *E*<10), to identify proteins with homologous motifs. Examples of homologous proteins retrieved from databases are presented.

Peptide	Homologous sequence	Example of homologous proteins	Biological activity	Identity (%)	*E*-value	Accession number
**4T1pep1 CPTASNTSC**	^2489^PTSSNSVVTS^2498^	No significant similarity found [*Mus musculus*]				
		Mucin-16 [*Homo sapiens*]	Reports show influence on cellular growth, differentiation, transformation, adhesion, invasion and immune surveillance	60	2.5	NP_078966.2
**4T1pep2 EVQSSKFPAHVS**	^1125^SKFPSHI^1131^	WD repeat membrane protein [*Mus musculus*]	Adaptor/regulatory modules in signal transduction, pre-mRNA processing and cytoskeleton assembly	71	0.014	AAK38746.1
	^1184^SKFPSHI^1190^	WD repeat membrane protein [*Homo sapiens*]		71	0.093	AAK38745.1
	^4651^QSTKFP^1656^	Mucin-16 [*Homo sapiens*]	Reports show influence on cellular growth, differentiation, transformation, adhesion, invasion and immune surveillance	83	0.26	NP_078966.2

## Discussion

TNBC is a highly aggressive subcategory of breast cancer and currently lacks well-defined molecular targets for effective targeted therapies [9, 34]. The 4T1 murine mammary carcinoma cell line has been shown to be an accurate xenograft model for stage IV of human breast cancer [35, 36]. Targeting approaches based on overexpression of specific molecules are often varied by cancer cell heterogeneity, which can lead to poor drug response. Although phage display has been applied for targeting breast cancer [37], the recognition of specific cell receptors for phage particle cloning and active targeting to the TNBC subtype are still scarce [4, 6]. Moreover, only one study has been reported regarding 4T1 targeting, supporting the need to find alternative targeting systems for this aggressive cancer subtype [13].

We report the selection of two novel peptides by phage display (using two different libraries), which revealed high binding efficiency and selectivity towards the 4T1 cell line. The selection from phage display libraries is driven by two main steps, (1) the screening step that enriches for clones binding to the desired target or any other physical moieties present; and (2) the amplification step, which consists on the infection of bacteria and secretion of enriched phage clones that have an advantage during any amplification step [38, 39]. In the first biopanning experiment using the C7C library, clones were sequenced between rounds and the retrieved peptide hits were analyzed. Although five different peptides were identified, a 100% enrichment of the 4T1pep1 (CPTASNTSC) was observed, thus this peptide was selected for further studies.

In the second biopanning experiment using the 12-mer library, phage amplification between rounds was performed due to the loss of phage titer in the previous biopanning protocol, individual clones from the last round were selected for DNA sequencing and the peptide sequences were retrieved. Pair-wise multiple alignments also showed a 100% similarity between all clones from the last round that were randomly selected, amplified and sequenced. However, it is common to obtain homology between DNA of clones from the same round. Indeed, variability may only be noted when 30 or more clones are sequenced or when clones from earlier rounds are also analyzed [40]. Nonetheless, it is important to reinforce that both peptides were accurately selected in two different and independent biopanning experiments, yielding the same frequency in the last biopanning rounds, hence confirming that they are interesting hits as novel peptides for the 4T1 cell line and that amplification between rounds may somewhat reduce binding efficiency, but not selectivity [30, 41].

Moreover, although biased sequences were minimized using a specific *E*. *coli*^+^ strain JM109, it is important to discard the possibility of non-specific targets, related to the materials used during the biopanning process, as well as to identify potential false positives. For this purpose, web-based tools, such as PepBank and SAROTUP, can be used to search for peptides that have been reported [42], or for peptides that bind unintended materials, respectively [27]. A preliminary analysis with PepBank showed no relevant data concerning similarity between the identified peptides and otherwise published peptides, which imposed a focused analysis and, on the other hand, supported the novelty of this study.

To further validate the binding ability of the selected sequences, proving our hypothesis, an immunofluorescence assay was performed. The results showed a positive binding of the selected peptides to the 4T1 cell line and, interestingly, to the human MDA-MB-231 cells. These results were also corroborated by a flow cytometry analysis, which clearly showed that there was no affinity to the control murine 3T3 cell line and that both 4T1pep1 and 4T1pep2 selectively bound the 4T1 cells. A significant binding capacity to the human MDA-MB-231 cell line was observed, which was expected, as many peptides for murine cell lines show highly conserved moieties or domains that closely resemble the corresponding human isoform and species cross-reactivity between selected peptides using phage display has been reported before [43]. Also, the 4T1pep1 showed a somewhat higher binding capacity as compared to the 4T1pep2, concerning both the murine and human cell lines. This finding was not surprising as peptides with cysteine terminal residues have generally demonstrated higher affinity to target cells than the linear peptides [44].

Small peptides identified by biopanning have the unique characteristic of binding to sites involved in many important functional interactions. These peptides are often capable of interfering with protein–protein interactions, as they share sufficient homology between the peptide and the ligand, allowing for target identification by a narrowed, but accurate receptor’ ligand sequence homology search [45]. Based on this, a multiple sequence alignment for a narrowed search of cancer-related or cancer putative proteins was performed for both peptides, leading to the identification of interesting targets. The narrowed search greatly reduced the number of artificial hits that would be generated randomly in the absence of an accurate search query. Hits were selected considering the use of whole cells in the biopanning experiments, enabling the identification of cell surface molecules that could be further used as diagnostic markers or therapeutic targets [19].

Sequence homology was found amongst two main significant proteins for both murine and human using the peptides 4T1pep1 and 4T1pep2. Regarding the first peptide, no similarity was found when retrieved against murine protein targets (*E*-values > 10). This fact may be due to the reduced peptide size (9 amino acids). For the same sequence, a human protein target Mucin-16 (or CA125) was found with 60% homology achieved with several matches considering the amino acid sequence PTSSNSVVTS. For the 4T1pep2, the best *E*-value was obtained for a murine or human WD repeat membrane protein family, which also showed 71% identity. Although it has been implied in the regulation of several cellular functions, signaling pathways and cell differentiation [46], the large size of this specific family makes it difficult to analyze a specific protein target for such a short peptide sequence. Nonetheless, it was compelling to observe that homology to the Mucin-16 was also found for this peptide. An overall identity of 83% and up to 6 amino acids overlap was obtained for the several matches that were shown throughout the N-terminus extracellular region of the protein, namely concerning the amino acid sequences QSSKFP and EVQSS.

Mucin-16 is a well characterized biomarker and its deregulation has been previously linked to cancer and inflammation, showing overexpression in breast, prostate, lung and pancreas cancer [47]. The N-terminus of Mucin-16 comprises a heavily O-glycosylated, non-tandem repeat domain of ~12 000 amino acids. These tandem repeat structures contain a high proportion of proline, threonine and serine (which constitute the PTS domain). These physicochemical properties are also shown for the selected peptides, which could have contributed to such homology. This hypothesis can be supported by the immunofluorescence and flow cytometry data, which show a similar trend in selectivity for both peptides. Mucin-16 has already been explored for breast cancer therapy, which makes it a promising target for the novel 4T1 peptide identified in this work [48, 49]. Future analysis underlying docking and binding experiments for this protein could be interesting to prove its role as a targeting receptor using these novel peptides.

Novel peptides and motifs identified by phage display are promising for application towards therapeutic moieties. Additionally, previous studies have shown that specific targeting of tumors not only diminishes side effects, but potentiates overall activity of classical chemotherapy agents and promotes translational medicine, potentially revolutionizing cancer therapy as it exists [16, 50, 51].

## Conclusions

In this study, we successfully identified two novel peptides - 4T1pep1 and 4T1pep2—through *in vitro* phage display biopanning against a specific TNBC murine cell line. To our knowledge, only one study reported a peptide targeting this specific cell line. The results showed a high enrichment of the selected peptides over various biopanning rounds and selective binding to both 4T1 mammary murine tissues and human MDA-MB-231 cell line. Altogether, this provides evidence that the peptides may have a translational application towards both human and murine targeting ligands. Moreover, through a homology search query, a promising mucin type isoform (Mucin-16) hit protein, which is involved in breast cancer progression, has been identified. Overall, the 4T1 peptides herein selected may serve as novel ligands that can potentially be explored for nano-based targeted delivery for TNBC therapy.
